# Artificial Intelligence-Based Teleopthalmology Application for Diagnosis of Diabetics Retinopathy

**DOI:** 10.1109/OJEMB.2022.3192780

**Published:** 2022-07-20

**Authors:** S. Ghouali, EM. Onyema, MS. Guellil, M A. Wajid, O. Clare, W. Cherifi, M. Feham

**Affiliations:** Faculty of Sciences and TechnologyMustapha Stambouli University 257229 Mascara 29000 Algeria; Department of Mathematics and Computer ScienceCoal City University 621825 Enugu 400104 Nigeria; Department of Mathematics and Computer ScienceCoal City University 621825 Enugu 400104 Nigeria; Adjunct Faculty, Saveetha School of EngineeringSaveetha Institute of Medical and Technical Sciences 584722 Chennai 602105 India; Faculty of Economics, Business and Management Sciences, MCLDL LaboratoryUniversity of Mascara 257229 Mascara 29000 Algeria; Department of Computer ScienceAligarh Muslim University 30037 Aligarh 202002 India; Department of Mathematics and Computer ScienceCoal City University 621825 Enugu 400104 Nigeria; InnoDev (Dev Software) Tlemcen 13000 Algeria; STIC Lab, Faculty of TechnologyUniversity of Tlemcen 173060 Tlemcen 13000 Algeria

**Keywords:** Deep learning, diabetic retinopathy, eye fundus images, tensorflow, artificial intelligence, smart health, IoT

## Abstract

Diabetic Retinopathy (DR) is one of the leading causes of blindness for people who have diabetes in the world. However, early detection of this disease can essentially decrease its effects on the patient. The recent breakthroughs in technologies, including the use of smart health systems based on Artificial intelligence, IoT and Blockchain are trying to improve the early diagnosis and treatment of diabetic retinopathy. In this study, we presented an AI-based smart teleopthalmology application for diagnosis of diabetic retinopathy. The app has the ability to facilitate the analyses of eye fundus images via deep learning from the Kaggle database using Tensor Flow mathematical library. The app would be useful in promoting mHealth and timely treatment of diabetic retinopathy by clinicians. With the AI-based application presented in this paper, patients can easily get supports and physicians and researchers can also mine or predict data on diabetic retinopathy and reports generated could assist doctors to determine the level of severity of the disease among the people.

## I. Introduction

The use of Artificial intelligence based health technologies holds the potential to improve healthcare services and medical emergencies. Over the past decade, a growing body of researches [1] have shown that deep learning, the branch of artificial intelligence that transforms data patterns into predictions, can be an advantageous method for a range of complicated tasks, including diagnosing multiple forms of the disease [2], speeding up drug development, and delivering precision treatment. Telemedicine is an excellent application of new information technologies in the medical field. It aims to improve access to healthcare and enhance e-health. This new medical practice method applies to each speciality and is linked through new technologies: the patient, health professionals, or several health professionals. Currently, telemedicine is known for its unprecedented growth with the evolution of component, which is the mobile phone that has completely revolutionized it. Although AI holds tremendous promise for improving diabetic retinopathy treatment, the distance between technological discovery and clinically practical technology adoption continues to be significant. Diabetic retinopathy is a health condition that affects eyes and it occur by damage to the blood vessels of the light-sensitive tissue at the back of the eye (retina). The treatment of the disease has not been what it should be and there is need for use of technology.

With the emergence of smart technologies like the one presented in this study, it is easier to detect and treat eye conditions before any loss of vision. In this study, we focused on diabetic retinopathy which has become a source of concern to global health systems.

Considering the dangers imposed by the disease, it has become more important than ever for people to get regular comprehensive dilated eye exam to avoid a major problem [3].

The use of smart health applications offers many benefits, including remote monitoring, medication recall and management, continuous retrieval of physiological data, patient location, detection of movement and shock when a person falls, diagnosis and early intervention for various types of diseases, understanding and, electronic consulting. Emerging technologies such as IoT, AI and blockchain are even making the health sector better [1], [4] clinical follow-up and remote patient monitoring with communicating measuring devices, supervision coordination and management of human resources, remote diagnosis and decision support are being made much easier by these technologies. IoT devices are helping health workers to connect with their patent and also identify possible approaches for treatments. The application presented in this study further proves the growing influence of technologies in the health sector. This paper presents AI-based solutions that would assist physicians and patients to diagnose or detect diabetic retinopathy early in order to start early treatment and reduce the severe effects and mortality rate due to the disease. Also, part of the problems that the outcome of this study will solve is to reduce the mortality associated with diabetic retinopathy in addition to improving treatment outcome. The work carried out in this context and the results obtained are gathered in several sections (I) Introduction, (II) M-health applications, (III) diabetic retinopathy application and the Medical Context to better understand this anomaly of Diabetic Retinopathy, (IV) The role of Artificial intelligence in this type of applications with the depth learning models for the treatment of DR images, (V, VI) Libraries and Tools, (VII) presentation of our application.

## II. M-Health Applications

Several contributions to health research have been widely expanded and cover areas such as heart disease [2], [5], [6], [21]–[24], diabetes [7], [8], obesity [9], smoking cessation [10] and care of the elderly and chronic diseases [2], [11], [70]. These medical specialities are mainly used in e-health and, more specifically, in surveillance, prevention, disease screening and advanced diagnostic services. In addition to all medical applications, there are also standard services in developing countries [12], where health facilities are often remote, inaccessible or non-existent. This is why mobile applications for health systems are increasingly used, developed and subject to significant developments. Research in this field attracts more and more interest every day and grows a range of influence areas. Most mobile systems handle several different types of communications. Whether for telephony and data management (GSM, GPRS) [13], synchronization (Infrared, Bluetooth), networks and the Internet (TCP/IP) [14], or messaging (e-mail, SMS, MMS), all these technologies requires a certain number of resources to be taken into account within the operating system itself. In the soft part, several APIs (Application Programming Interface) allow the management of these communications [15]. The continued growth and penetration of mobile devices in many parts of the world could be leveraged upon to enhance access to healthcare and response to health emergencies [16]. Health authorities, particularly those in developing countries must do more to maximize the benefits of mobiles in the health sector.

## III. Diabetic Retinopathy Application

**Fig. 1.**
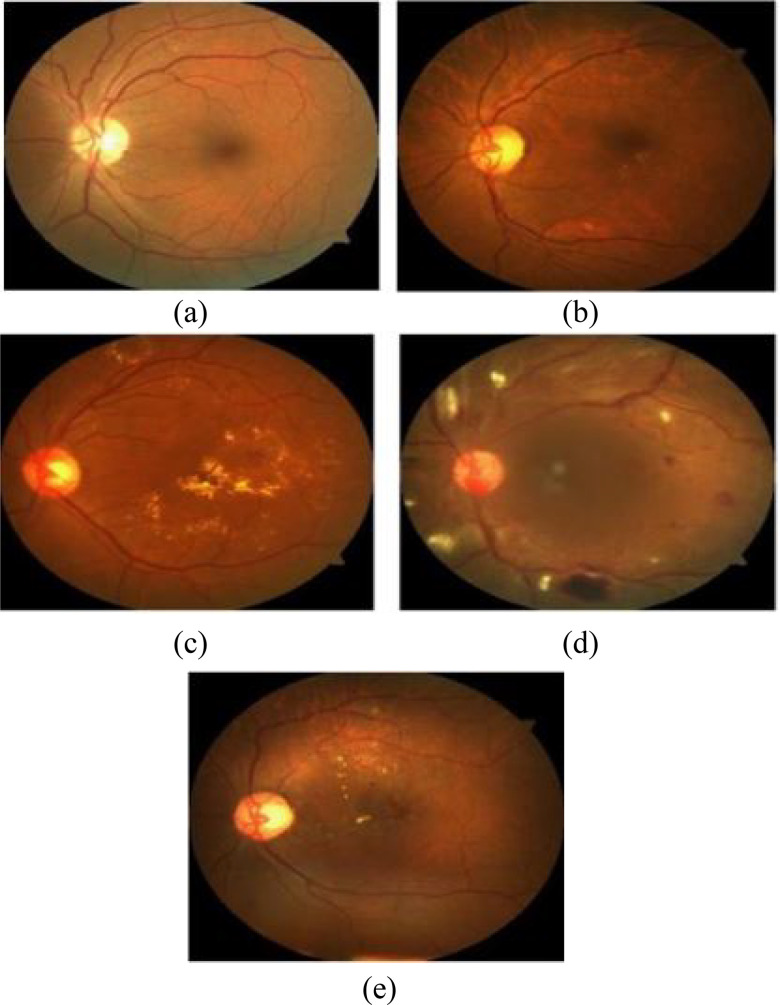
Different DR levels.

Many telemedicine systems developed worldwide can detect various retinal diseases. Also, research has shown that diabetes is one of the major diseases responsible for retinal blindness [17], [18]. Most importantly, the number of people with diabetes over the age of 64 will exceed 82 million in emerging countries with limited health conditions by 2022, and nearly 40 million people will live mainly in remote areas of developed countries blinded by cataracts and diabetic retinopathy [19], [20]. Fig. 1 shows retina images with different DR levels.

**Fig. 2.**
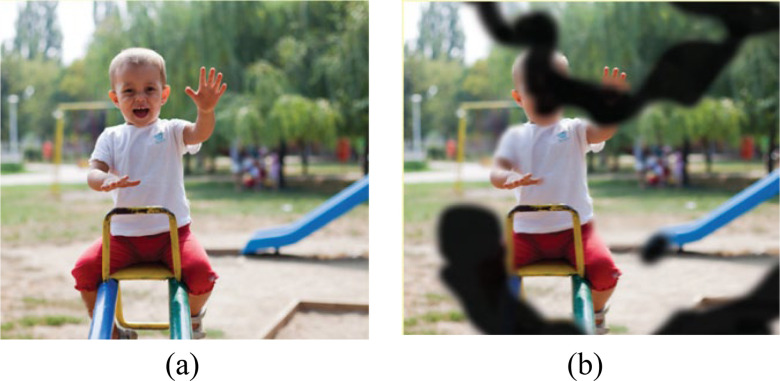
Effect of diabetic retinopathy: (a) Normal vision, (b) Vision with DR. (National Institute of Health).

**Fig. 3.**
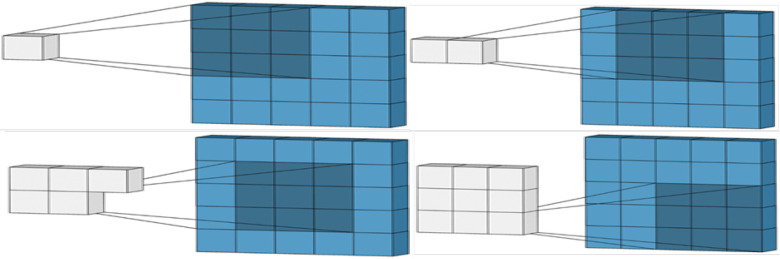
Matrices convolution.

**Fig. 4.**
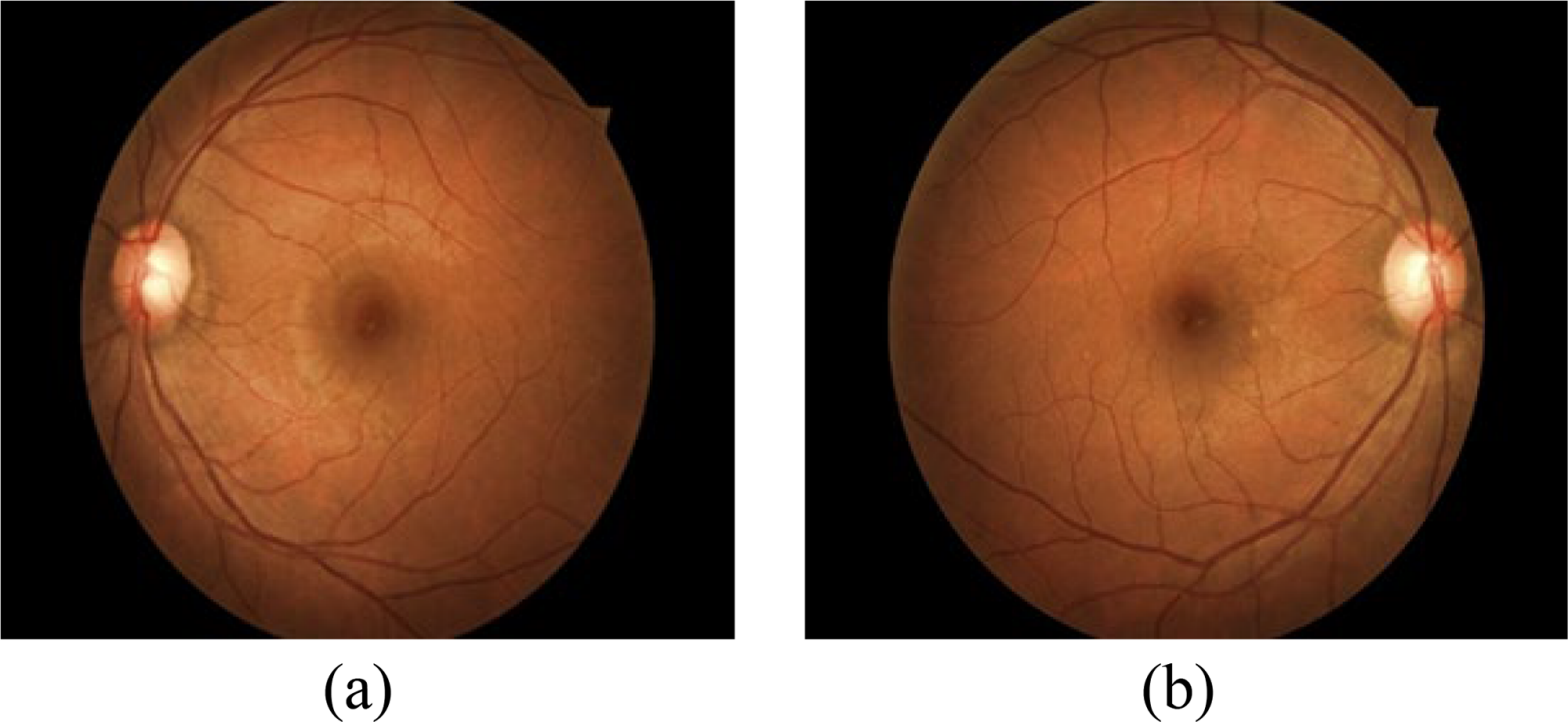
(a) Right eye fundus; (b) Left eye fundus.

**Fig. 5.**
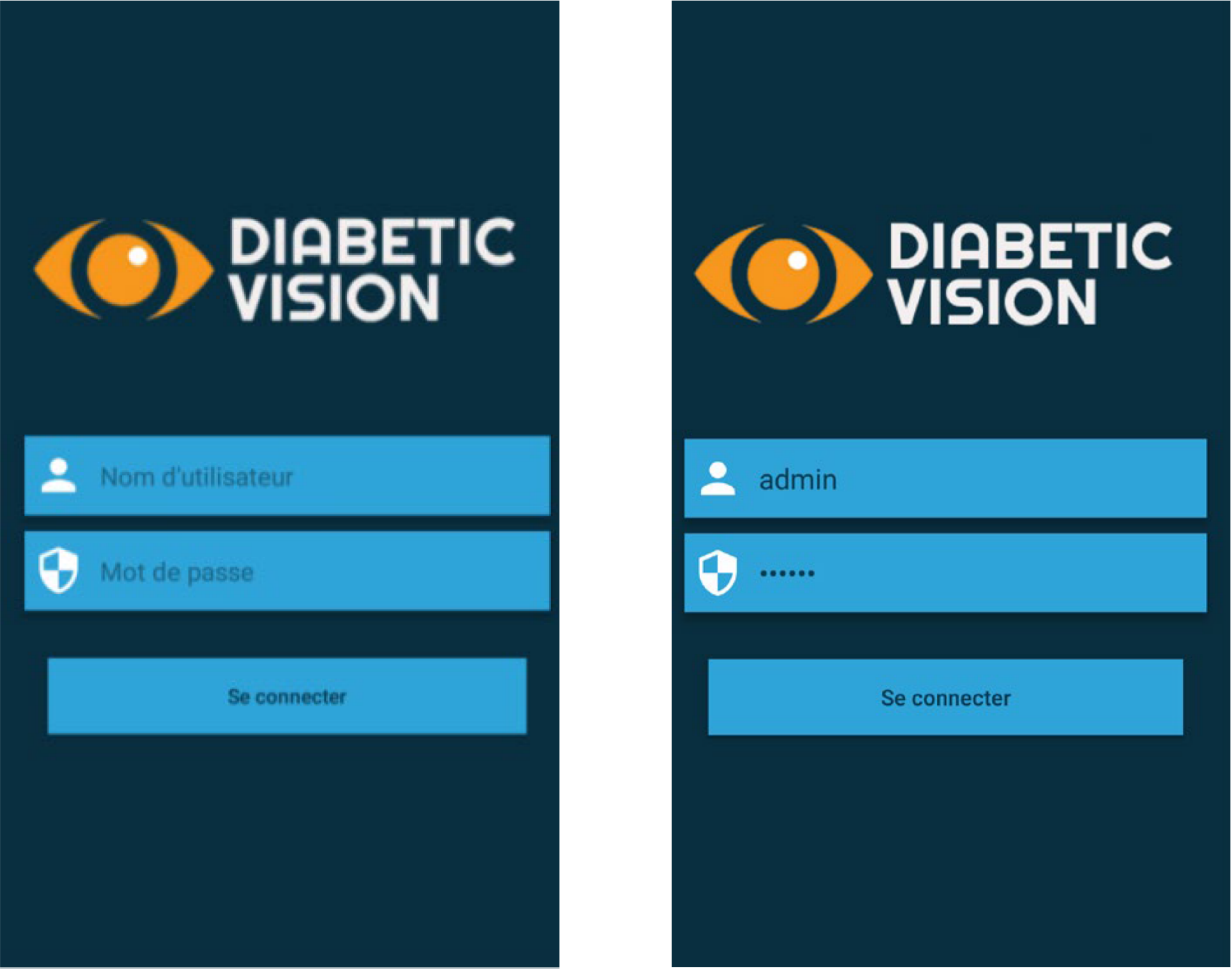
Authentication.

**Fig. 6.**
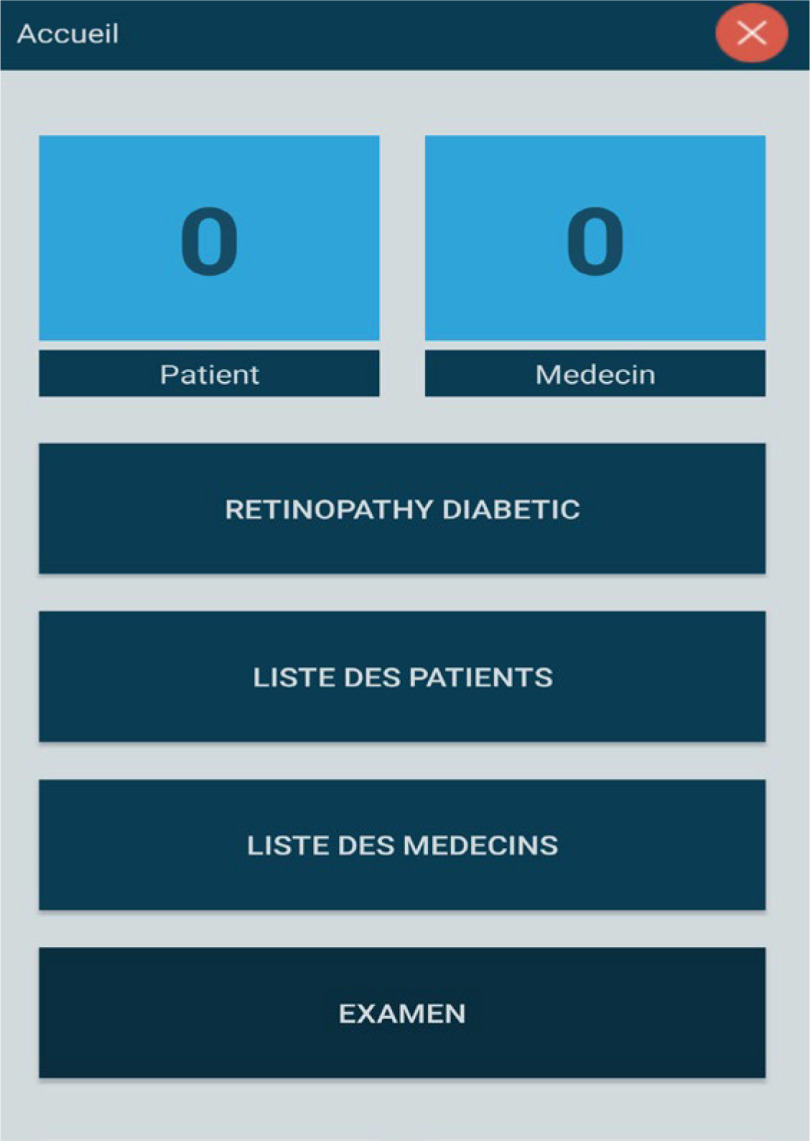
Menu screen.

**Fig. 7.**
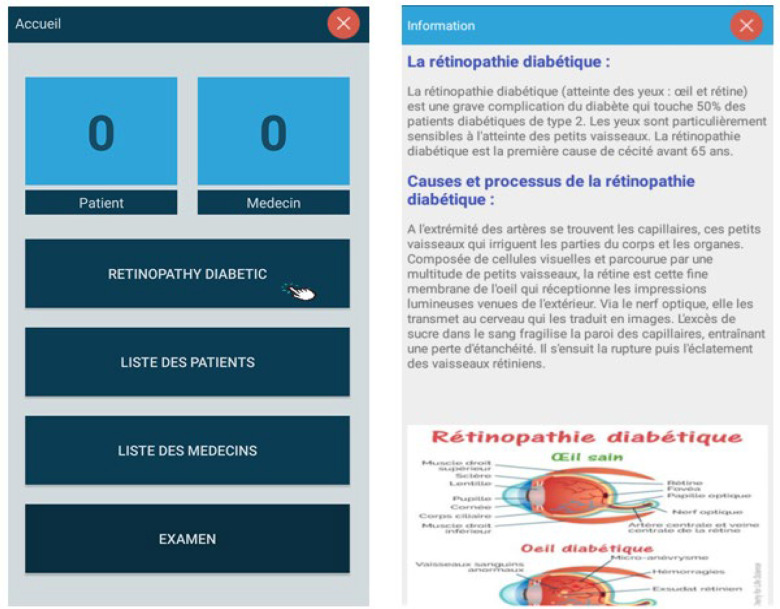
Retinopathy diabetic information.

**Fig. 8.**
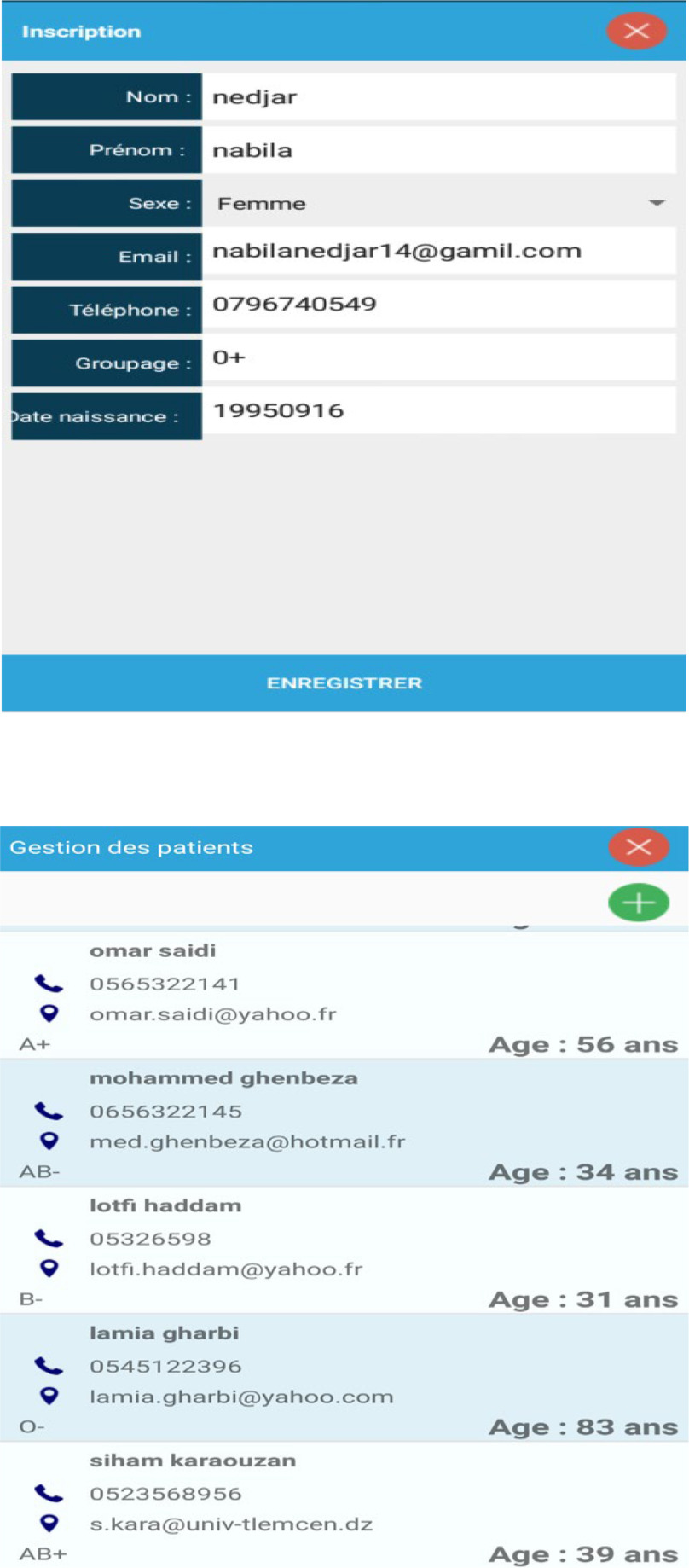
Patients lists.

**Fig. 9.**
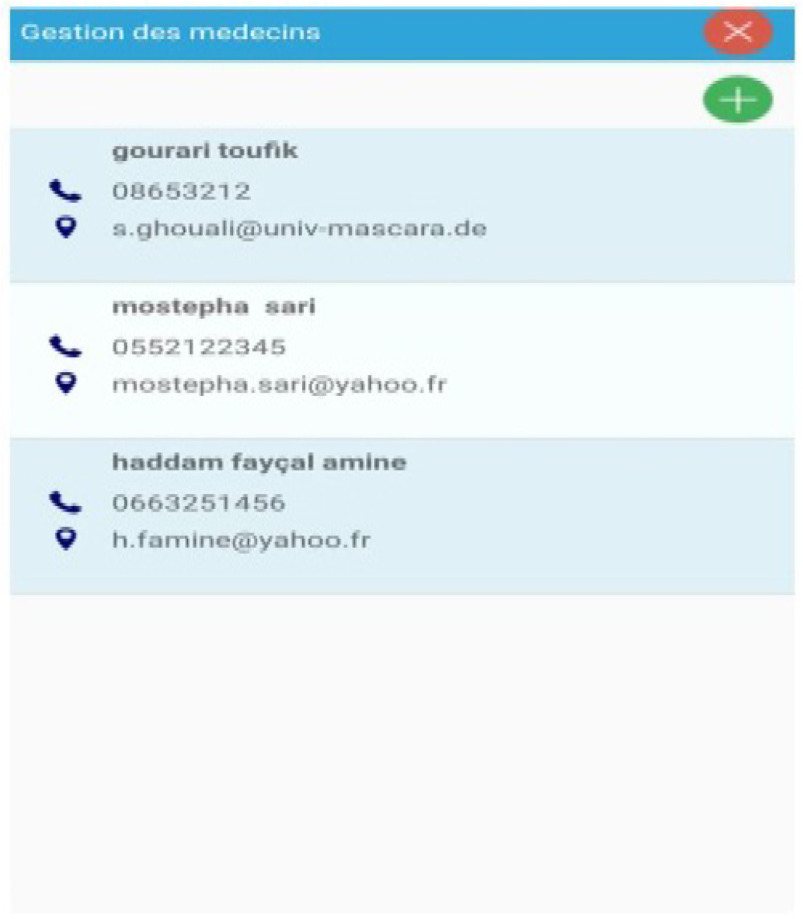
Doctors lists.

**Fig. 10.**
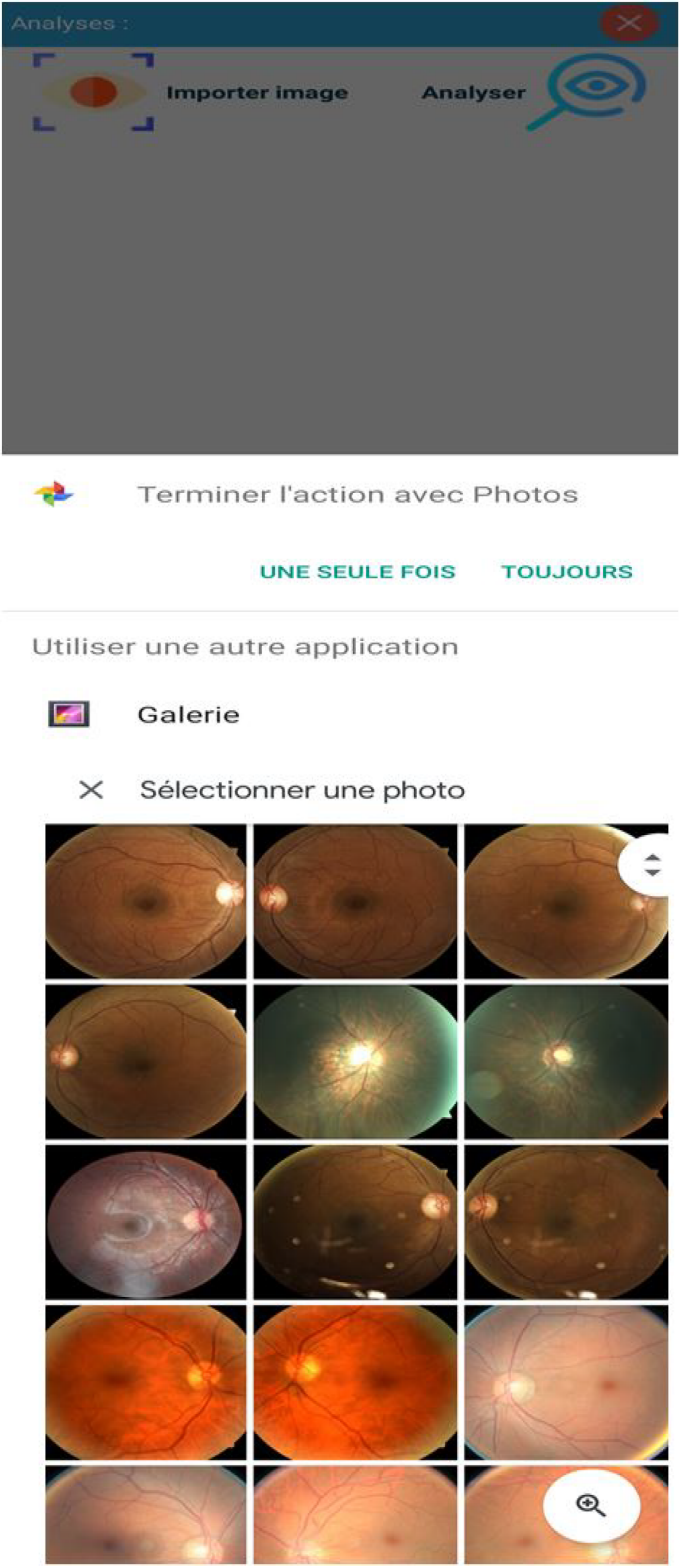
RD database.

**Fig. 11.**
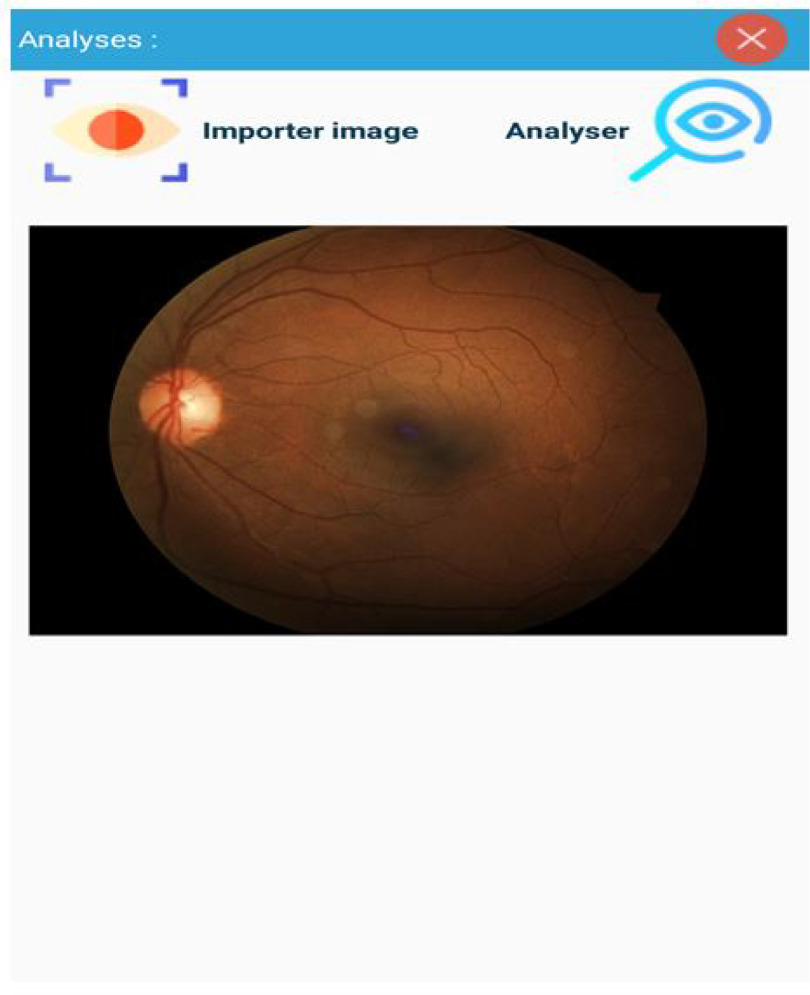
Choice of picture to be examined.

**Fig. 12.**
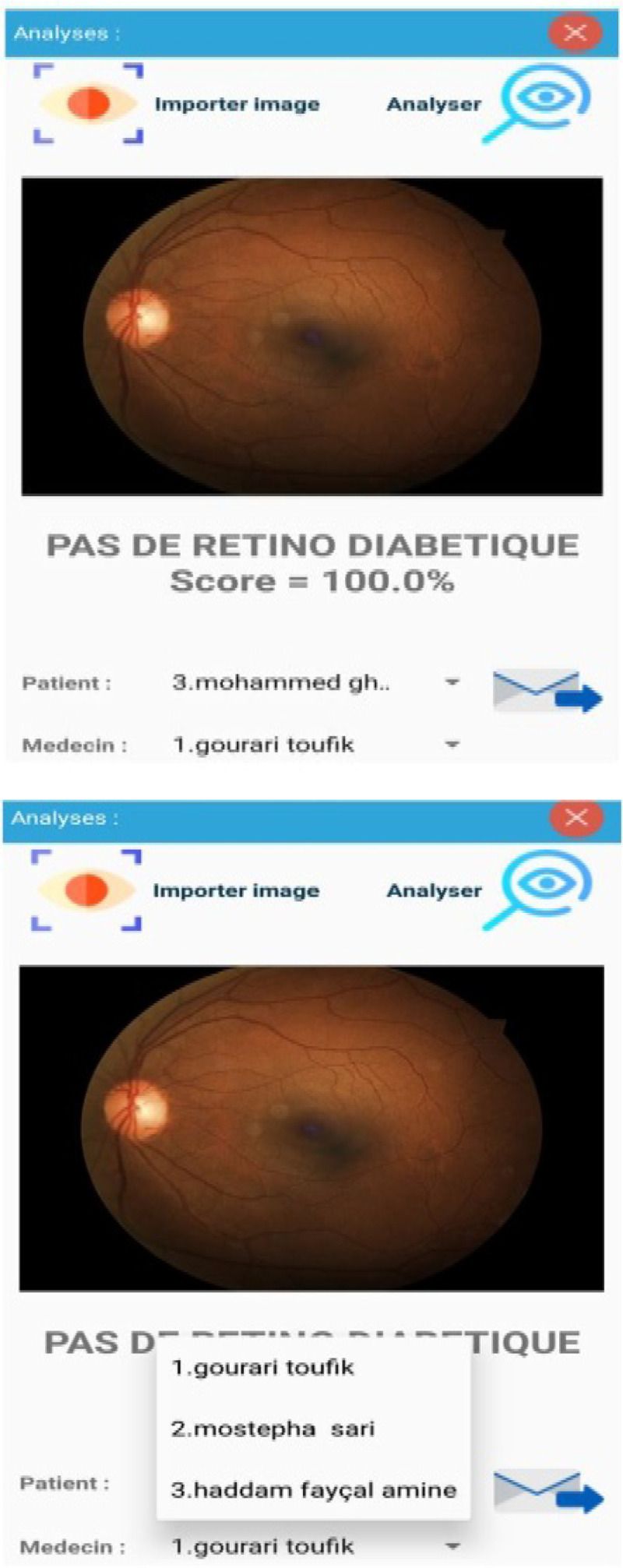
Examination Results.

The Fig. 1 represents how the condition of patients with diabetic retinopathy can worsen if not detected early enough and treated. This is one of the things that the present study intended to solve. The condition tends to worsens and becomes more severe as the stage advances without treatment. As can been seen, at stage A, the patient can still see with little obstructions, but as it gets to stage B, C, D and E, it gets darker. The stage E is the worst and can lead to total blindness.

The need to mitigate the growing dangers associated with Diabetic Retinopathy motivated us to develop a smart teleopthalmology application for diagnosis of diabetic retinopathy. The application would go a long way to bridge the gap that exists in the diagnosis of DR and patient-provider communication. It will enhance efficiency and management of the healthcare system, particularly as it relates to diabetic retinopathy. Physicians will be able to identify and manage cases at easy and treat patients more speedily. The application will not only provide data, but can be pooled together to study and predict future trends in DR.

Digital health-care systems such as the one presented in this paper could leverage the use of IoT and big data to seamlessly connect patients and providers across diverse health-care systems.

### A. Cataract (Crystalline)

A Cataract is the opacification of all or part of the focal point, a uniting focal point situated inside the eye [25], [26] which can lead to a decrease in vision.

### B. Glaucoma (Optic Nerve)

Glaucoma is an infection of the optic nerve that associates the eyeball to the cerebrum [27]–[31].

### C. Diabetic Retinopathy DR

Diabetes has several factors, including genetic and environmental factors, characterized by a permanent increase in blood sugar. Faced with an incredible rise in the number of patients, scientific researchers are now talking about the epidemic of a diabetes. The complications of diabetes type 2 are the risk of this disease because it can damage the heart, kidneys, arteries, nerves and eyes [32]–[34], [71]–[74].

Research has shown that about 300 million people suffer from diabetic retinopathy (230 million in developing countries and 70 million in industrialized countries), and this figure could well double by 2025 [35]–[38].

Indeed, DR is a silent condition for many years due to damage to small vessels. Per good clinical practice recommendations, only regular screening can enable early diagnosis and treatment [39], [40]. Figure 2 shows the different effects of diabetic retinopathy in human.

Extensive epidemiological studies provided a better understanding of the retinal complications of DR. About 10% of people with diabetes have vision problems [36].

However, it is estimated that early detection and treatment of retinal damage could prevent more than 95% of visual acuity declines in people with diabetes. A few factors, such as blood pressure and proteinuria, also assume a job in the improvement and development of retinopathy. Epidemiological studies in industrialized countries cite it as one of the four leading causes of visual impairment in the general population and the leading cause of blindness in people under the age of 65 [41], [42].

This condition is not noticed for many years, and it only becomes symptomatic at the complication stage. Delayed and deferred treatment is the primary source of vision misfortune and is preventable with appropriate screening and treatment [43]–[46]. This disease can be diagnosed early and treated only through regular examination, as blindness and visual impairment associated with diabetic retinopathy are primarily preventable with laser treatment, whose effectiveness has long been proven [41], [42].

The objective of screening for diabetic retinopathy is to prevent visual impairment due to retinopathy by early identification of the disease and implementing an appropriate intervention.

## IV. Artificial Intelligence

The use of AI related devices is helping physicians to treat eye related diseases and many other health challenges. Different AI algorithms are being used to detect visual examples straight forwardly from pixel pictures with insignificant and minimal pre-processing. In recent years, several convolutional architectures have developed enormously, including AlexNet [47], VGGNet [48], GoogleNet [49], [50], ResNet [51], ResNext [52], SENet [53], DenseNet [54] have been proposed.

For recurrent neural networks, there are LSTM [55], Bi-RNNNN [56], GRU [57], Memory network [58] and Attention network [59]. However, how to parallelize the RNNN is still a significant problem under active investigation [53], [59], [60]. At the beginning of this section, we will present some of the impressive libraries such as Keras, Scikit-learn, NumPy, SciPy, Matplotlib, Pandas, Seabornet, TensorFlow [61]. We will present TensorFlow's complete concept, which represents our choice to develop our application.

## V. Learning Libraries

### A. Keras

Keras is a library that makes it much easier to create these in-depth learning solutions. In a few code lines, we can create a model that implements hundreds of conventional code lines [62].

### B. Scikit-Learn

This is another popular Python library for automatic learning [63].

### C. NumPy

NumPy is another incredible python library for automatic learning and intensive computing [64].

### D. TensorFlow

TensorFlow is an automatic data-learning library. A team created it called the Brain Team and developed by Google in 2015 [65]. This is all about the most popular python libraries for in-depth automatic learning. Therefore, from these libraries, we can choose them according to our objectives. As part of our work to develop our application to detect the disease Diabetic Retinopathy, we will use the TensorFlow library.

The development of mobile applications for in-depth learning has become a revolutionary field with vast potential and applications. It is a subset of Machine Learning which is a vast field. The two main factors of success are:

• Access to computing power: GPUs (graphics processing units) have made it possible to process huge matrices [66] quickly;
• Access to vast volumes of data (Big Data): we have more data than ever before.

In 2015, it was transformed into a library based on much better applications [67]. Several features of Tensor Flow explain its popularity. It is one of the interactive cross-platform programs that are very stable, unlike other in-depth learning libraries. As a result, this is among the reasons that we choose Tensor Flow when developing our application. There are essential characteristics: Open source, has API's for Matlab, C++ and Java, reactive constructive, flexible, and easily trainable (But it requires a more or less powerful GPU graphics card’ from NVIDIA 1050’ for a good learning experience), availability of statistical distributions, it supports threads, asynchronous calculations and queues.

Several Tensor Flow automatic learning applications exist in the world around us, such as sentiment analysis, Google translates, text synthesis and image recognition by leading companies around the world such as Airbnb, eBay, Dropbox, Snapchat, Twitter, Uber, SAP, Qualcomm, IBM, Intel, Google, Facebook, Instagram and even Amazon.

Some of the main applications include Voice recognition systems, image and video recognition, autonomous cars and a summary of texts for researchers. There are many TensorFlow images recognition models such as QuocNet, AlexNet and Inception. Now, they have taken a new step by publishing the code for Inception-v3, which represents the latest image recognition, model.

• To read and write an image document, we must import the File class [import java.io.File;],
• To process errors, we use the IOException class [import java.io.IOException;],
• To maintain the image, we make the BufferedImage protest because we use the BufferedImage class [import java.awt.image.BufferedImage;], this method queries and uses RAM to store a processed image,
• To reproduce the image reading composition activity, we will import the ImageIO class [import javax.imageio.imageio.ImageIO;]. This class has static strategies for browsing and composing an image.

This is an essential part of Java image processing because pixels are the smallest unit of an image that consists of four parts: Alpha (linearity measurement), Red, Green and Blue and in short (ARGB). The estimate of the considerable number of segments is in the vicinity of 0 and 255 both overall. Zero means the absence of segments, and 255 indicates that the feature is entirely present. In this part of Java image processing, we will create a watermark and apply it to an info image to generate content and use it to an image. We will use java.awt.Graphics Bundle. The textual style and nuance of the content are linked using the classes’ java.awt.Color and java.awt.Font. In the next section, we will present a foreword to several morphological tools that can be integrated into systems/applications and play an essential role and may be sufficient for learning and DR detection. In the following, we will present our application DIABETIC VISION which makes it possible to exploit the screening of diabetic retinopathy. In the beginning, we describe the working methodology adopted to develop it. Then, we will present our programming tools used, which depend essentially on mathematical morphology. Finally, we will explain the sequence of our application and how it works.

## VI. Analysis Tools

### A. Android Operating System

There are different types of operating systems for mobile phones and each of them has its benefits and demerits. Therefore, it is not easy to choose the platform that best suits a company or person's purpose. Then, we will present the way Android is chosen and offer the different system tools to create and develop our application. Android is an open-source operating system for tablets, smartphones and mobile devices. It emerged from a consortium of 34 companies (in 2008), developed by Google on November 5, 2007, and named the Open Handset Alliance or OHA. Its objective was to find the compatible solution to compete with Apple with iPhone OS, Microsoft with Windows Mobile and Nokia with Symbian (Jean Kruger 2009). Due to its performance and development efficiency, Android is the market leader for smartphones and tablets. The Fig. 3 below shows Android's global market share.

### B. Convolution Matrix

Convolution is the treatment of a matrix by another one which is called kernel. The Convolution Matrix filter uses a first matrix which is the Image to be treated.

### C. Kaggle Database

Kaggle conducted a DR identification challenge in 2015 [68]. The California Healthcare Foundation funded the California Health Achievement Award Competition. The Kaggle database contains 88, 702 different colour picture backgrounds as seen in Fig. 4.

The clinicians have evaluated each image for the presence or absence of DR with a scale of 0 to 4 according to ETDRS standards [69]. Kaggle DR is the largest DR classification database available. There are a certain number of non-classifiable fundus as well as others of poor quality so it is necessary to take into account these parameters.

### D. Images Transmission

This part of the project works to find the best way to send the images through our application. There are many possibilities; we can transfer the image by Email. Our analysis allowed us to better understand the work of the main protocols that will be used for application development, leading to the writing of the analysis report.

## VII. Result and Discussion

In this paper, we developed an AI-Based Smart teleopthalmology application for diagnosis of diabetic retinopathy. The application is Android based and could be used to facilitate the early detection and screening of diabetic retinopathy. It facilitates mhealth and more-effective treatment and management of diabetic retinopathy.

Physicians and patients can leverage on the various features of the application to interact better and also exchange smart medical data. The application can be used to carry out diagnosis via TensorFlow deep learning and results can be sent to the patients via email by the physicians or hospital. The data generated from the application can be helpful in curbing the rising cases of eye-related diseases.

The ability of Artificial intelligence applications as demonstrated in this study would make it easier for early discovery, treatment and forecasting of likely pandemics and suggestion of possible treatments. The application would enhance data recording and mining regarding diabetic retinopathy and also increase the awareness about the disease and the need for authorities to take preventive measures to eradicate it. The application presented in this paper would enhance routine understanding of the changing patterns of diabetic retinopathy by experts with a view to provide better and real-time assistance to physicians and patients.

### A. Execution

In order to benefit from the developed application, the user must follow these steps:

Step 1: Install the application and activate all permissions.
Step 2: After installing the application then opening it, the Splash screen appears and authentication is required (Username and Password). Figure 5 shows the screenshot for the login page of the app.
Step 3: After setting the correct password and login, in a few seconds, the Menu screen appears. In this menu, there are headings such as (Retinopathy Diabetic, List of patients, doctors, and the more important section of the Retinopathy examination) as depicted in Fig. 6 and 7.
Step 4: In the Menu screen, click on the first item:
Step 5: By clicking on the Patient List, the user will have to complete the list and all the data will be sent to an internal SQLite database that does not require servers or an internet connection.

In the same way, the list of ophthalmic doctors is replicated: Figure 8 shows the list of patients who have registered in the app while Fig 9 shows that of doctors who can respond to patient's queries.

Step 6: An automatic filling of the database, which will then be viewed so that contacts can be made either for our patients or for the treating ophthalmologists.
Step 7: This is the most important part where we will do the background image test for a patient X.
Step 8: Sending the mail to the desired ophthalmologist for different server boxes (Yahoo, Hotmail, Gmail...)
Step 9: Perform several tests for our patients even with dark eyes.

The Figure 10, 11, and 12 shows the background image test for a given patient X. These images are often analyzed by doctors after examinations.

### B. Benefits of the Smart Teleopthalmology Application

• It enhances the use of smart health applications and quick detection of diabetic retinopathy.
• Detection of Diabetic Retinopathy via TensorFlow from the Kaggle database with an instant report.
• Sending a detailed message (Email) containing the patient's name, the detailed report of the retinopathy result and even the background image.
• Improved access to medical services
• Enhances mhealth and physician-patient interaction
• Facilitates medical data records, health delivery and cost reduction
• Enhances diagnosis and timely treatment.
• Improved patients medical experience and access.

### C. Tests and Results

The results of the classification/performance of the algorithm blocks implemented in our application can also be evaluated using parameters well known in the literature, such as precision, specificity and sensitivity, associated with each type of pathology. These three parameters are calculated as follows:

\begin{align*}
& \text{Precision} = \frac{{{\rm{Total\ number\ of\ eye\ bases\ detected\ DR}}/\text{NoDR}}}{{{\rm{Total\ number\ of\ eye\ bases}}}} \tag{1}\\
&\text{Specificity} = \frac{{{\rm{Total\ number\ of\ NoDR\ eye\ bases\ detected}}}}{{{\rm{Total\ number\ of\ NoDR\ eye\ bases}}}} \tag{2}\\
&\ \text{Sensitivity} = \frac{{{\rm{Total\ number\ of\ DR\ eye\ bases\ detected}}}}{{{\rm{Total\ number\ of\ DR\ eye\ bases}}}} \tag{3}
\end{align*}

As part of the Kaggle database, we took samples processed by our application DIABETIC VISION. The results obtained are presented in table 1 and 2 the following tables:

**TABLE 1:** Comparison of the Results Between the Kaggle Database and Our Application Diabetic Vision

	NODR	DR
KAGGLE	6190	494
DIABETIC VISION	5655	457

**TABLE 2:** Results

	**DIABETIC VISION**
**Precision**	91.44%
**Specificity**	91.35%
**Sensitivity**	92.51%

### D. Performances

Android Profiler is one of the tools integrated in Android Studio 3.0 and above. It replaces the Android Monitor tools. The event chronology displays events related to user input including keyboard activity, database changes, and analyses and even screen rotations. These timeline views which include graphs for CPU, memory, network consumption, represent the test results of the smart teleopthalmology application for diagnosis of diabetic retinopathy.

We can clearly see the optimized consumption of our application whether it is on the graphics side (42.8 mb), CPU (35%), RAM (<128 mb) and even on the network side (273 kb/s). When sending the mail, this makes the smart teleopthalmology application for diagnosis of diabetic retinopathy more stable and can be more efficient. This study supports the growing studies which affirmed that use of AI-driven applications can indeed increase chances of treatment and survival [75], [76].

## VIII. Conclusion and Future Work

The use of AI would go a long way to enhance smart healthcare which would be better accessible to the public. The implementation of smart healthcare system with the health of AI, IOT and other emerging technologies would improve healthcare and medical emergencies. Embedded telemedicine applications are emerging and have doubled compared to mobile devices using smartphones. Telemedicine aims to partially mitigate medical deserts and to improve the efficiency of care. Therefore, the examination of DR is currently a real big problem. Our work is part of the diagnostic assistance for the analysis of background images using telemedicine tools and techniques. In addition, improving the quality of patient care with the increase in the number of people with diabetes is being reviewed annually.

The presented AI-based smart teleopthalmology application for diagnosis of diabetic retinopathy is an Android application that facilitate early detection and screening of diabetic retinopathy. It supports smart health care and prompt diagnosis and treatment of diabetic retinopathy, thereby helping to curb the mortality rates related to the disease. As a perspective to this work, we will soon be able to implement deductions on heavy automatic learning models by integrating a fixed-point model with the TensorFlow Lite which has the role of optimizing the processing side. We conclude that AI, IoT and blockchain and many other emerging technologies would shape the future of healthcare. Thus, there is the need for stakeholders in the health sector to equip themselves with modern skills that support the implementation or adoption of smart health care systems in line with the trend. In the future we will work on AI-based devices for diagnosis of myopia and Apea syndrome.
